# TV advertising and dietary intake in adolescents: a pre- and post- study of Chile’s Food Marketing Policy

**DOI:** 10.1186/s12966-021-01126-7

**Published:** 2021-05-04

**Authors:** Melissa L. Jensen, Francesca R. Dillman Carpentier, Linda Adair, Camila Corvalán, Barry M. Popkin, Lindsey Smith Taillie

**Affiliations:** 1 Department of Nutrition, Gillings School of Global Public Health, University of North Carolina, Chapel Hill, NC USA; 2 Global Food Research Program, Carolina Population Center, Chapel Hill, NC USA; 3 Escuela de Nutrición, Universidad de Costa Rica, San José, Costa Rica; 4 Hussman School of Media and Journalism, University of North Carolina, Chapel Hill, NC USA; 5 Instituto de Nutrición y Tecnología de Alimentos, Universidad de Chile, Macul, Chile

**Keywords:** Food marketing, Food advertising, Food environment

## Abstract

**Background:**

The first phase of a comprehensive marketing policy was implemented in Chile in 2016. The policy restricted child-directed marketing of foods and beverages considered high in energy, total sugars, sodium or saturated fat (“high-in”). The objective of this study was to examine the role of high-in TV food advertising as a mediator in the association between policy implementation and consumption of high-in foods and beverages between 2016 and 2017.

**Methods:**

Dietary data were from 24-hour diet recalls collected in 2016 and 2017 in a cohort of 12–14 y children (*n* = 721). Television use was assessed concurrently and linked to analyses of food advertisements on broadcast and paid television to derive individual-level estimates of exposure to high-in food advertising. A multilevel mediation analysis examined direct and indirect effects of the policy through advertising exposure.

**Results:**

Following the policy implementation, high-in advertising exposure was significantly reduced (*p* < 0.01). High-in food intake decreased in adolescents with lower levels, but not higher levels, of high-in advertising at baseline. We did not find evidence of mediation by changes in high-in ad exposure.

**Conclusions:**

Adolescents’ exposure to high-in TV advertising decreased after the 2016 implementation of the Chilean Food Labeling and Marketing Law. However, evidence that changes in advertisement mediated dietary changes was not found. Further research is needed to understand how marketing changes will relate to dietary changes after full implementation of the law and in the long term.

**Supplementary Information:**

The online version contains supplementary material available at 10.1186/s12966-021-01126-7.

## Background

Marketing of unhealthy food and beverages influences food preferences, attitudes and consumption among youth [1–5], and it is a global health concern, because most of the promoted products are high in energy, saturated fat, sugars, or sodium, and are of little nutritional value [6]. Regulations to reduce children’s food marketing have been implemented around the globe, although the majority of these have been voluntary industry-led initiatives, which have largely been found to be ineffective [7]. Governmental regulations are less common, with few systematic evaluations of the effects of these policies [7, 8]. Studies of how changes in advertising due to government restrictions affects dietary intake in a naturalistic setting are lacking. Furthermore, most of the policies enacted to date have focused on younger children, mostly due to evidence suggesting that children do not recognize the persuasive intent of marketing until close to age 12 [9]. This is worrisome because adolescents have unique developmental and social characteristics that also make them vulnerable to the effects of food marketing [10].

In Chile, the government implemented the first phase of its Law of Food Labeling and Advertising (Law 20,606) [11–13] in 2016. There were several major policy changes that were enacted along with the marketing restriction component of the regulation: foods that exceeded energy and saturated fat, sugars or sodium thresholds (hereafter high-in) are prohibited from being sold in schools, and they are also subject to a front-of-package warning label [12]. Given that these changes occurred simultaneously, the evaluation of changes in food intake that are due specifically to the marketing restriction is complex. Past evaluations of the Chilean policy have quantified changes either on marketing exposure [14],[15] or on the purchasing changes in specific food groups, such as sugar-sweetened beverages (SSBs) [16]. After the first phase of the regulation, adolescents’ exposure to televised food advertising of high-in products decreased significantly by an average of 58 % [14]. However, it is unknown how these changes in advertising might be related to changes in dietary intake beyond changes that occurred as the result of time (aging) or other policy components. Mediation analysis is a helpful tool because it allows for examination of intermediate pathways or mechanisms through which an intervention may have an effect [17]. Because global recommendations often include adopting a package of policy actions to create healthier food environments [18], mediation can help evaluate single components of such policies, when the policies target the same longer-term outcomes (dietary intake, food purchases, health status, among others).

Another important gap is understanding whether there are specific subgroups of the population that might be more or less affected by a policy restricting TV advertising. For example, a higher baseline preference for taxed foods [19], as well as socioeconomic status [20] have been associated with larger declines in taxed purchases following Mexico’s 8 % nonessential food tax. However, it is unknown whether differential effects would also be the present in a TV food advertising restriction. In particular, it is unclear whether adolescents who had a higher level of pre-policy advertising exposure would experience stronger effects of the marketing policy on dietary intake.

This longitudinal study uses data on adolescents’ exposure to TV food advertising and dietary intake to examine the role of TV food advertising as a mediator in the association between policy implementation and consumption of high-in foods and beverages between 2016 (pre-policy) and 2017 (post-policy). Our focus is on television (TV) advertising, because TV is an important promotional channel [4] and used widely in Chile [21]. Chilean children age 13–17 spent 116 min (1.9 h) per day watching television in 2018 and 130 min (2.17 h) watching TV per day in 2017 [22]. In addition, TV was the largest ad medium in 2018 (43 %, or about 266.4 million in US dollars of ad spending given to free TV) [23]. We hypothesized that (1) the policy would be negatively associated with TV food advertising, (2) that intake of high-in foods (our main outcome of interest) in 2017 would be lower than intake in 2016, and (3) the difference in intake between 2017 and 2016 would be mediated by exposure to TV advertising.

## Methods

### Policy context

In the first phase of the marketing restriction component of the Chilean law, marketing of high-in foods—foods exceeding government-defined thresholds for added total sugars, saturated fats, sodium, and energy was prohibited to children under 14 years of age [11, 13]. This includes marketing material placed in content (e.g., advertising in television programs) that was either intended for children < 14 yo or attracted audiences where 20 % or more of its audience included children < 14 yo. A new regulation was implemented in 2018, which extended marketing restrictions of high-in foods in cinema and TV to a 6 am to 10 pm period. The focus of this paper is on the first phase of Law 20,606.

### Study design and participants

We used data from two waves (2016 and 2017) of the Growth and Obesity Cohort Study (GOCS), a longitudinal cohort of children who were 12 to 14 years old in 2016. GOCS participants were recruited from low- and middle-income neighborhoods in the southeastern area of Santiago, Chile in 2006 when they were 3–5 years old. Recruitment strategies and inclusion and exclusion criteria are described elsewhere [24, 25]. Our sample was comprised of 721 participants. The ethics committee of the Institute of Nutrition and Food Technology and the University of North Carolina – at Chapel Hill approved the study protocol, and we obtained written informed consent from parents or legal guardians of participants, as well as an assent from adolescents prior to conducting the interviews.

### Data Collection

#### Advertising exposure

A detailed explanation of how individual-level advertising exposure measures were estimated for this study has been previously published [14]. Participants reported weekly hours and channels of television use, with items adapted from the Global Weekly Estimate of children’s television viewing [26] and channels viewed. Reports were used to calculate hours the participant viewed specific television channels during different times of day on weekdays and weekends. A separate concurrent content analysis of TV advertisements was done in 2016 and 2017 to identify the amount and nutritional quality of food and beverage ads among eight highly viewed TV channels [27], including their food and beverage category, regulation status, and time and channel of airing.

The regulation status for each advertised product was assigned by linking products appearing in the ad with nutrition facts panel (NFP) data collected pre-regulation in 2015–2016 and post-regulation in 2017 [28, 29]. Trained nutritionists reviewed and assigned a regulation status to each product based on the nutrition content of the product and the thresholds of the Chilean law. The thresholds were 350 kilocalories of total energy, 22.5 g of sugars, 6 g of saturated fats, and 800 milligrams of sodium for solid foods (per 100 g) and 100 kilocalories of total energy, 6 g of sugars, 3 g of saturated fats, and 100 milligrams of sodium for liquids (per 100 mL). For this paper, packaged products exceeding these nutrient thresholds are called high-in products, and ads featuring any high-in product are designated high-in ads.

#### Dietary intake

Participants completed a 24-hour dietary recall, which was administered by trained dietitians using a multiple-pass method assisted by a computer software developed for such purposes. A second 24-hour recall was collected in 20 % of the sample each year (2016 and 2017). Serving sizes of common Chilean food and beverages were assessed with a food atlas that included use of images such as bowls, plates, mugs, and glasses [30]. Nutrient values were calculated from the USDA Food and Nutrient Database [31], as well as information from the NFP of packaged products in Chilean supermarkets. The NFP data were collected from high- and low-income supermarkets during the study period, using a standardized photographic methodology [28].

An initial step in the analysis was to update the food composition table from which nutrient information for diet data was derived with the NFP data that was collected for each corresponding year. Therefore, the updated food composition table contained NFP values for two different time periods, pre- and post-regulation. To calculate children’s nutrient intakes resulting from recalls collected in 2016, the linkage was done to the NFP values of 2015–2016; likewise, to calculate nutrient intakes for 2017, the linkage was done to the NFP values of 2017. This update was important, so that dietary intakes could reflect nutrient values of products in the Chilean food supply, which might have been reformulated in response to the regulation. The linkage was done at the product-specific level; and in cases in which the NFP value for the exact product was not available, the most similar product was used (for example, if the child reported grape-flavored drink, but only strawberry flavor was available, the linkage was done to this product).

To assign high-in status of foods consumed, we adopted a practical step-wise approach. First, we identified all the individual foods that were reported throughout the 24-hour dietary recalls. Then, these were manually coded as packaged/non-packaged based on the detailed name of product (for example, searching for a brand name or packaging description). Once these products were identified, high-in status was given to those that based on the packaging information exceeded the thresholds for energy, saturated fats, total sugars, and/or sodium. High-in status was assigned per year, so that products could become non-high-in due to reformulation or threshold changing. Note that the nutrient cut points only apply to products with added nutrients of concern (for example, a 100 % fruit juice with no added sugar is not considered high-in, according to the Chilean law).

### Measurements

#### Outcomes: Food consumption

Our primary outcome variable was consumption of high-in foods and beverages, expressed both as absolute quantity energy intake (kilocalories) and energy-adjusted (percent of total daily energy intake). We present both absolute and energy-adjusted, because energy requirements increase with growth, and therefore caloric intake is expected to increase with time [32]. Consumption of five key food groups and three nutrients of concern were considered secondary outcomes, and only used in analysis when assessing baseline association of marketing with food consumption. The five food groups of interest were: ready-to-eat breakfast cereals, salty snacks, sweets and desserts, sugar sweetened beverages, and milks and yogurts (drinkables and edibles). These five food groups are energy-dense products commonly marketed on television to children and adolescents globally [6] and also highly advertised in Chile [27]. Our food consumption variables were derived from the first dietary recall of all study participants, except in cases in which this recall was unreliable (according to dietitians collecting the data) or not a usual intake day (according to study participant) in which cases the second recall available was used in its place (82.5 % of recalls were usual).

#### Food advertising

Participants’ exposure to food advertising was measured in weekly minutes. The weekly number of minutes of exposure to advertising was determined for products specifically high in: (i) energy, (ii) total sugars, (iii) sodium, and (iv) saturated fats. Because some ads exceeded the thresholds for multiple nutrients of concern, these categories were not mutually exclusive. We also calculated an overall number of weekly minutes of exposure to advertising featuring at least one product with any exceeded threshold.

#### Covariates

Covariates included age (in years), sex at birth (dichotomous), mother’s education level (less than high school, completed high school and above high school), marital state (married/living with partner or other), home ownership (yes/no), and day of dietary recall (weekday/weekend).

### Statistical analyses

First, we examined the association between high-in TV advertising and daily dietary intake (overall high-in consumption, high-in consumption by food groups, and daily intake of nutrients of concern and food groups) at baseline. To assess these associations, we categorized participants into three exposure groups with the use of tertiles: low (0-3.1 min), medium (3.1–9.5 min), and high (9.5–31.8 min) high-in advertising exposure. Tertiles were considered more appropriate given that the distribution of the exposures of interest were skewed, as well as to ensure similar sample size within each comparison group. We used multivariable regression models adjusting for the aforementioned covariates and post-hoc pairwise comparisons to examine differences in the main outcomes by groups compared to our reference group (low exposure). This analysis was done for the entire sample available at baseline (*n* = 721).

To model possible direct and indirect effects of the policy through TV food advertising, we conducted a mediation analysis. We restricted this analyses to participants with complete dietary and advertising data at follow-up (*n* = 679, 94.1 % of baseline sample), because this analysis required pre- and post- policy data. Participants who had been interviewed during the week as opposed to during the weekend were more likely to remain in the sample at follow-up **(Table **S1), no other differences were observed for nutrients, advertising exposure or covariates.

Regression models were used to test whether exposure to TV advertising mediated the association between the pre-post policy implementation period and consumption of high-in products (both in absolute intake [kcal] and energy adjusted [percent calories]). A total of five mediation analyses were performed, each using one of the following mediators (all in weekly minutes): TV advertising with products high in (1) total calories, (2) total sugars, (3) saturated fats, (4) sodium, and (5) any of the thresholds. Policy implementation was dichotomous, with 1 = post-policy, and 0 = pre-policy. All models controlled for the aforementioned covariates. To account for inflation of Type I error, a familywise error correction was applied and an alpha value of 0.01 (0.05/5 mediators = 0.01) was considered significant.

**Fig. 1:**
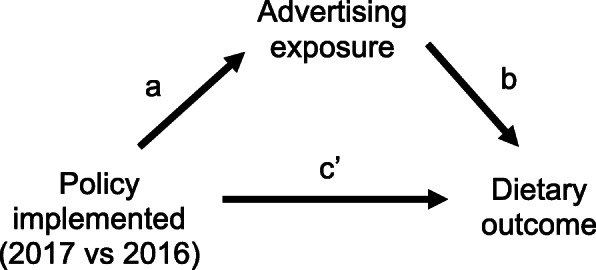
Mediation pathway between policy implementation, advertising exposure and dietary outcomes

We estimated indirect effects by using the product of coefficient method [17, 33]. To account for the longitudinal nature of the data, a two-level model was fit (Stata GSEM), and a random intercept was included in each equation at the individual level. For each mediation model, the following associations were tested (Fig. 1): (i) the association between policy implementation and each advertising exposure variable (a-coefficient); (ii) the associations between each advertising exposure variables and dietary outcomes, controlling for policy implementation (b-coefficient); and (iii) the direct effect of policy implementation, accounting for the advertising variable (c’-coefficient). The indirect effect of policy implementation through ad exposure in each mediation analysis was calculated by multiplying the a and b coefficients, and then presented as the percentage of the total effect mediated (ab/(c’ +ab))*100. To estimate the statistical significance of the a*b coefficient, 99 % confidence intervals con corresponding p-values were assessed. Mediation exists if a*b is different from zero.

To examine whether the association between policy implementation and food consumption differed by baseline levels of advertising we used two-level mixed models with random intercepts at the individual level, adjusting for covariates. An interaction term between baseline level of advertising (low, medium, and high, as determined by tertiles) and policy period (2016 vs. 2017) was added. All analyses were conducted using STATA 16.0 software (StataCorp LP, College Station, Texas, 2019).

### Sensitivity analyses

We performed four additional analyses. (i) To examine the influence of outliers, all analyses were repeated excluding outliers for total caloric intake (*n* = 10 and *n* = 18, for baseline and longitudinal analyses, respectively). (ii) Given that the range of minutes of high-in advertising for the high exposure group was wide (9.5–31.8 min), we also explored two alternate groupings (four and six categories) to confirm that baseline relationship at a higher level of advertising was not being obscured by the tertile classification. (iii) Because at baseline female participants had average higher exposure to advertising, and lower energy consumption, we also conducted the baseline and mediation analyses stratified by sex. (iv) Given differential changes in high-in food consumption by baseline levels of high-in advertising exposure, we explored full mediation models stratified by these baseline levels of high-in ad exposure.

## Results

### Participant characteristics

Study participant characteristics at baseline are shown in Table 1. The mean age was 13.6 ± 0.4 and 50.5 % of our sample were female. The mean exposure to high-in ads was 7.5 ± 6.6 per day, and the mean consumption of high-in foods and beverages was 555 ± 385 kcal, which was 29.7 ± 18.5 % of daily caloric intake.

**Table 1:** Baseline sample characteristics of study participants and their mothers (*n* = 721)

	mean	sd	n	%
**Child characteristics**				
Age (years)	13.6	0.4		
Female			364	50.5
TV viewing time (hours per week)	14.8	10.9		
Exposure to high-in ads (minutes per week)				
Any “High In” ad	7.5	6.6		
High calorie	3.2	2.9		
High total sugar	4.8	4.3		
High saturated fat	1.8	1.7		
High sodium	0.7	0.7		
Consumption of high-in food and beverages				
Per capita absolute intake (kcal)	555	385		
Per capita energy adjusted intake (% kcal of total)	29.7	18.5		
Percentage consuming			709	98.3
Per consumer absolute intake (kcal)	572	434		
Per consumer energy adjusted intake (% kcal of total)	30.7	18.5		
**Primary caregiver characteristics**				
Education level (%)				
Less than high school			213	29.5
High school complete			342	47.4
More than high school			166	23.0
Married or living with partner			391	54.2
Family owns home			410	56.9

### Baseline associations of food consumption and TV advertising

Compared to adolescents with low high-in ad exposure, those with high exposure at baseline had higher absolute intake of high-in foods and beverages (kcal); however, this difference did not reach statistical significance (Table 2), and the relationship was not step-wise. Total energy intake was highest among high exposure adolescents, when compared to low advertising exposure (*p* < 0.05), and no appreciable differences were observed in nutrient intake. For food groups, adolescents with at least some advertising exposure consumed on average less high-in ready-to-eat breakfast cereals, but more high-in sugar sweetened beverages compared to children with no exposure (*p* < 0.05).

**Table 2:** Daily dietary intake by baseline levels of ad exposure (in tertiles)

	Low exposure (*n* = 241)	Medium exposure (*n* = 240)	High exposure (*n* = 240)
	0.0-3.1 min	3.1-9.5 min	9.5-31.8 min
**Overall high-in foods**^**1,2**^			
Consumers: *n (%)*	*236 (97.9)*	*233 (97.1)*	*230 (95.8)*
Absolute energy (kcal)	561 ± 28	527 ± 28	576 ± 28
Energy adjusted (% kcal)	31.4 ± 1.2	28.5 ± 1.2	29.3 ± 1.2
**Daily intake (energy and nutrients)**			
Daily energy (kcal)	1739 ± 41	1804 ± 40	1865 ± 41*
Total sugars (grams)	97.4 ± 3.6	98.7 ± 3.6	104.3 ± 3.6
Total sugars (% energy)	22.4 ± 0.6	22.0 ± 0.6	22.2 ± 0.6
Saturated fat (grams)	20.4 ± 0.7	20.6 ± 0.7	21.3 ± 0.7
Saturated fat (% energy)	10.4 ± 0.2	10.0 ± 0.2	10.0 ± 0.2
Sodium (mg)	2413 ± 73	2422 ± 73	2552 ± 73
Sodium (mg/1000 kcal)	1399 ± 30	1363 ± 30	1386 ± 30
**Food groups (high-in)**			
**Ready-to-eat breakfast cereals**			
Consumers: *n (%)*	*46 (19.1)*	*30 (12.5)*	*30 (12.5)*
Absolute energy (kcal)	32 ± 4	18 ± 4*	17 ± 4*
Energy adjusted (% kcal)	1.8 ± 0.2	1.2 ± 0.2	0.9 ± 0.2*
**Salty snacks**			
Consumers: *n (%)*	*47 (19.5)*	*42 (17.5)*	*55(22.9)*
Absolute energy (kcal)	64 ± 12	67 ± 12	68 ± 12
Energy adjusted (% kcal)	3.3 ± 0.6	3.4 ± 0.6	3.6 ± 0.6
**Sweets and desserts**			
Consumers: *n (%)*	*159 (66.0)*	*159 (66.3)*	*149 (62.1)*
Absolute energy (kcal)	218 ± 17	197 ± 17	216 ± 17
Energy adjusted (% kcal)	12.1 ± 0.9	10.7 ± 0.9	10.9 ± 0.9
**Sugar-sweetened beverages**			
Consumers: *n (%*)	*133 (55.2)*	*141 (58.8)*	*139 (57.9)*
Absolute energy (kcal)	107 ± 10	110 ± 10	136 ± 10*
Energy adjusted (% kcal)	6.1 ± 0.5	5.9 ± 0.5	6.9 ± 0.5
**Milks and yogurts**			
Consumers: *n (%)*	*49 (20.3)*	*52 (21.7)*	*40 (16.7)*
Absolute energy (kcal)	29 ± 5	35 ± 5	28 ± 5
Energy adjusted (% kcal)	1.8 ± 0.3	2.1 ± 0.3	1.6 ± 0.3

^1^ Model adjusted for age (years) and sex of child, maternal education, home ownership, marital state, and day of week.

^2^ Values are mean ± SE, unless otherwise noted. *P < 0.05 for pairwise comparison with referent group (low exposure).

### Mediation analyses

The implementation of the policy had a significant effect on each type of high-in TV advertising exposure (a-coefficients), ranging from a mean decrease of 0.2 min/week for high sodium ads (99 %CI -0.3, 0.0) to a decrease of 4.6 min/week (99 %CI -5.7, -3.5) for any high-in ad (Table 3). Policy implementation did not have a significant total effect on consumption of high-in foods, neither in absolute intake (-53 kcal, 99CI: -147, -41) or energy adjusted (-3.7 %, 99CI: -7.4, 0.0). When examining the indirect effect, or mediating effect of “high-in” advertising exposure, none of the five models showed a significant indirect effect between policy implementation and high-in consumption through advertising (ab coefficient), however, all were in the expected direction.

**Table 3:** Mediation analyses displaying associations between policy implementation (2017 vs. 2016), advertising exposure, and consumption of overall high-in foods (*n* = 679; 1,358 observations)

	a-coefficient^2^		b-coefficient		c’-coefficient Direct Effect		ab Indirect (mediation) Effect		Proportion of total variance accounted for by ab path ^5^
	(policy→ ads)		(ads→ intake)		(policy→ intake)				
	β^3^	(99 % CI)	β	(99 % CI)	β	(99 % CI)	β	(99 % CI)	
**Absolute quantity (kcal)**^**1,3**^									
Any “High-in” ad	-4.6*	(-5.7, -3.5)	4	(-2, 10)	-37	(-135, 61)	-16	(-44, 12)	30.4 %
High calorie	-2.3*	(-2.8, -1.8)	8	(-6, 22)	-35	(-134, 64)	-18	(-50, 14)	34.2 %
High sugar	-3.0*	(-3.7, -2.3)	5	(-5, 14)	-39	(-137, 59)	-14	(-42, 15)	26.1 %
High fat	-1.3*	(-1.6, -1.0)	16	(-14, 46)	-32	(-133, 70)	-21	(-61, 18)	39.9 %
High sodium	-0.2*	(-0.3, 0.0)	31	(-18, 79)	-48	(-142, 46)	-5	(-14, 4)	9.4 %
**Energy adjusted (% kcal)**^**1,4**^									
Any “High-in” ad	-4.6*	(-5.7, -3.5)	0.0	(-0.2, 0.3)	-3.6	(-7.4, 0.3)	-0.1	(-1.2, 1.0)	3.0 %
High calorie	-2.3*	(-2.8, -1.8)	0.1	(-0.5, 0.6)	-3.5	(-7.4, 0.4)	-0.2	(-1.4, 1.1)	4.9 %
High sugar	-3.0*	(-3.7, -2.3)	0.0	(-0.4, 0.4)	-3.7	(-7.5, 0.2)	0.0	(-1.1, 1.1)	0.1 %
High Fat	-1.3*	(-1.6, -1.0)	0.1	(-0.9, 1.1)	-3.6	(-7.5, 0.4)	-0.1	(-1.4, 1.2)	3.0 %
High sodium	-0.2*	(-0.3, 0.0)	0.4	(-1.5, 2.3)	-3.6	(-7.3, 0.1)	-0.1	(-0.4, 0.3)	1.7 %

^1^Analyses adjusted for child’s sex and age, mother’s education level, marital state, home ownership, and day of recall

^2^ Expressed in minutes of advertising per week (a-coefficient reflects policy effect on advertising)

^3^ Total effect of policy on consumption of “high-in” products in kcal = -53 kcal [99CI: -147, 41] *p* = 0.146

^4^ Total effect of policy on energy adjusted consumption of high-in products = -3.7 % kcal [99CI: -7.4, 0.0] *p* = 0.011

^5^ ab/[(ab) + c’] *100

^*^p < 0.01.

### Change by baseline level of advertising

**Fig. 2:**
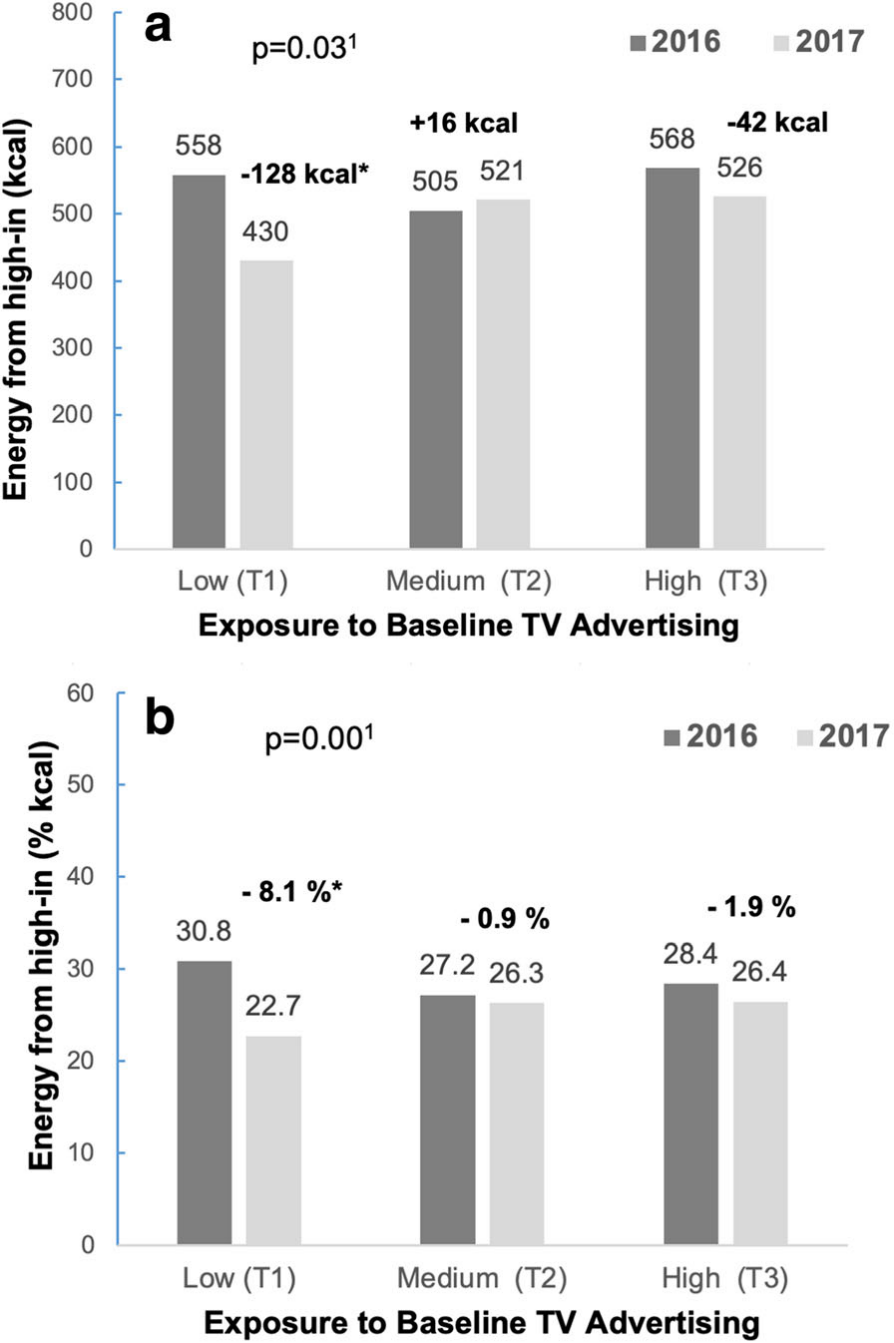
Participants (n = 679; 1,358 observations) consumption of high-in foods pre- and post-policy by baseline levels of high-in TV advertising. Panel a: absolute intake in kcal. Panel b: energy adjusted (% kcal). Estimated using mixed model with individuals as a random effect, adjusting for study covariates. Wald test for interaction (year*baseline ad exposure)* indicates *p* < 0.05

Figure 2 displays changes in consumption of high-in foods by baseline levels of advertising exposure, in absolute intake (Panel A) and energy adjusted (Panel B), respectively. Results from a Wald test for the interaction term indicated that there were significant differences in these changes both when expressed as absolute intake of high-in (kcal) and energy adjusted (% kcal). Adolescents with low levels of advertising at baseline decreased their consumption of high-in products (*p* < 0.05); the differences for adolescents in the medium and higher exposure groups were not statistically significant.

### Sensitivity analyses

Excluding outliers for total caloric intake, did not change our main conclusions for the baseline associations analyses **(Supplementary **S2), mediation analyses (**Table **S3), or pre-post changes by baseline levels of high-in advertising (**Fig. **S1). Exploring alternate groupings for high-in advertising when estimating baseline associations with high-in food consumption did not provide evidence that a step-wise or linear relationship was present (**Table **S4 and **Table **S5). Therefore, we considered more appropriate to maintain the tertile grouping, which ensures larger and similar sized samples in subgroups. When stratifying baseline analyses by sex (**Table **S6), the association between high-in advertising and lower consumption of high-in breakfast cereals became significant only among boys, however, most other associations remained the same. The sex-stratified mediation analyses (**Table **S7) suggested that the total effect of the policy was larger among girls compared to boys (5 % kcal decrease compared to 3 % kcal). However, because these differences might be partly due to differing energy requirements by sex, and that the direct and indirect (mediated) effects did not differ by sex, we present our main mediation analyses for the total non-stratified sample. Finally, stratifying our mediation models by baseline levels of high-in ad exposure (**Table **S8) evidenced that despite participants with lower high-in ad exposure at baseline displaying larger reductions in high-in food consumption, this decrease is not driven by high-in advertising (b- and ab- coefficients not largest among this group).

## Discussion

Our study found that after the initial implementation of the Chilean Law of Food Labelling and Marketing, adolescents experienced a significant decrease in their exposure to high-in TV advertising. We did not find evidence of mediation by changes in high-in ad exposure and therefore, our results suggest that factors other than the decrease in exposure to advertising might be driving the changes in consumption of high-in products (our main outcome of interest). In fact, changes in consumption of high-in products were only observed in adolescents with the lower levels of advertising at baseline. Our results are, to an extent, expected, given the complexity of the mechanisms of food marketing effects. Because these effects range from awareness of products and brands, to changes in attitudes and preferences, and interact with physiological and contextual influences [34], it is possible that the decrease in high-in ads after one year post-policy will reflect in dietary changes in the longer-term.

We believe that the lack of an association between high-in TV food advertising and high-in consumption in our study specifically, could be explained by at least two important considerations. First, was that high-in foods consumed and captured as our outcome might not completely match high-in advertised foods. Although we used the same definition for high-in products in ads and consumption, differences might exist in brands and subgroups consumed vs. advertised. Second, we were unable to determine to what extent product reformulation had an effect on results. Products might have been reformulated before our follow-up assessment of advertising [35], and in this case continued to be advertised to the child, but would not have been captured as high-in advertising to which the child was exposed to. Further research is needed to continue to understand whether being exposed to less TV advertising affects dietary intake in the longer term.

When comparing our results to those of a preschool children cohort, [36] adolescents decreased high-in consumption to a lesser extent (95 kcal [99CI: −138, -50] in children, compared to 53 kcal [99CI: -147, 41] in adolescents). Note that this is the total difference when comparing pre- and post- policy high-in calorie consumption, and the change was not statistically significant. Adolescents eating behaviors might be less influenced by parental purchasing decisions compared to preschool children, and their food preferences more strongly established, which makes it less likely their consumption would change in a 1-year time span. Furthermore, the 1-year energy requirement increase in adolescents is slightly more than that of preschool children [32] given developmental needs, and therefore they are consuming more food overall, which translates to their relative decrease being smaller. Finally, adolescents might be more likely to be exposed to advertising online, which might have diminished to an extent the effect of reductions in TV advertising on diet.

The first phase of Law 20,606 did not restrict TV advertising during all times of the day, and only banned ads that were child-directed, in the techniques used (i.e. cartoons, childhood references) or the program aired. That is, adolescents were not a specific target for this first stage of implementation, and despite this, they significantly decreased their exposure to high-in ads post policy. It is unknown whether a decrease in exposure to high-in ads would have also been the case for older adolescents (our sample was 12–14 y), since marketers could have shifted their targeting to older age groups given the scope of the first phase of the Law. We anticipate that the extended law will have a larger potential to affect adolescents, given that ads are restricted between 6 a.m. and 10 p.m. Another consideration is that adolescents are more likely than children to snack on highly advertised products, such as sweet desserts, salty snacks and soda [24], which highlights the importance of this policy. In Chile, children and adolescents (2–19 y) consumed on average more energy from ultra-processed foods (37 %) when compared to adults (22–28 %) and older adults (17 %). Furthermore, the association between ultra-processed food consumption and added sugars was stronger among children and adolescents [37].

An interesting and unexpected finding of our study was that adolescents with the lower levels of exposure to ads at baseline, were those whose food consumption decreased, both in absolute and energy adjusted quantities. Rather than reflecting an intrinsic policy mechanism, a possible explanation is that adolescents with lower levels of exposure to ads at baseline were also more likely to have health-conscious behaviors, making them more responsive to other components of the Chilean policy, such as the warning labels and the school food restrictions.

An important consideration moving forward is whether and how Chilean adolescents will be protected from other forms of marketing that might not be considered child-directed under the current regulation. For example, music celebrity endorsements for food and beverage products are common [38] as are sponsorships for sporting events [39], which might affect adolescents.

TV use has decreased in recent years among this age group [21], whereas smartphone use has increased. This highlights the importance of considering digital marketing and its potential effects on adolescents diets. Newer forms of marketing via social media and product placement tend to hide their persuasive intent [9]. The Food Marketing Defense model states that four conditions are necessary to defend against the influence of unhealthy food marketing: awareness of the marketing stimuli, understanding of how one is affected by marketing, ability and cognitive resources to resist, as well as an interest and desire to resist [40]. Therefore, if an individual cannot recognize the persuasive intent of the marketing, it becomes harder to defend against its effects [9].

The ability to assess individual level exposure to unhealthy TV advertising was a strength of this study, and to our knowledge, only a few studies have done so in children [41, 42] none of these in adolescents. Another key strength was the use of a longitudinal design and the mediation analyses to assess the association of advertising with subsequent dietary intake.

We wish to acknowledge several limitations of this study. Firstly, our primary outcome was restricted to packaged high-in foods, given that it was necessary to have the nutrition facts panel of products to determine their regulation status. Therefore, we might be underestimating the amount of unhealthy foods consumed (for example, an ice cream cone from an ice cream shop would not be captured in our outcome). Second, because all our data were self-reported, measurement error is a possibility, both in our TV advertising exposure and in our dietary data. Third, one 24-hour dietary recall is not representative of usual dietary intake, as it is a snapshot of an individual’s diet at one point in time. In an effort to account for this limitation, we asked participants whether their intake was a usual day, and considered this variable when selecting the primary dietary recall for analysis (83 % of recalls reported as usual). Fourth, while content analyses of TV ads covered eight highly viewed TV channels in Chile, a number of adolescents in our sample viewed channels not included in the advertising analyses [14]. Therefore, adolescents in Chile might be exposed to more high-in advertising than that estimated in our study.

## Conclusions

The implementation of the initial phase of the Chilean Law of Food Labelling and Marketing was associated with significant decreases in the exposure to TV advertising in Chilean adolescents. However, evidence of mediation by changes in high-in ad exposure was not found and decreases in consumption of high-in foods were only observed for adolescents with lower levels of baseline advertising. Continued monitoring of how overall marketing restrictions relate to dietary changes as the Law progresses to further stages is warranted.

## Supplementary information

**Additional file 1: Table S1.**Comparison of baseline sample (2016) with and without post-policy (2017) data. **Table S2.**Daily dietary intake by baseline levels of ad exposure excluding energy outliers (*n*=715). **Table S3.** Associations between policy implementation (2017 vs 2016) and consumption of overall regulated packaged foods accounting for mediation by advertising exposure excluding energy outliers (*n*=666; 1,332 observations). **Table S4.** Overall high-in consumption at baseline by levels of advertising categorized in four groups. **Table S5. **Overall high-in consumption at baseline by levels of advertising categorized in six groups. **Table S6.** Daily dietary intake by baseline levels of high-in ad exposure (in tertiles) and stratified by sex (*n*=721). **Table S7.** Associations between policy implementation (2017 vs 2016) and consumption of overall regulated packaged foods accounting for mediation by advertising exposure and stratified by sex (*n*=679; 1,358 observations). **Table S8.** Associations between policy implementation (2017 vs 2016) and consumption of overall regulated packaged foods accounting for mediation by advertising exposure and stratified by baseline levels of advertising(*n*=679; 1,358 observations). **Figure S1.** Participants (*n*=666; 1,332 observations) consumption of HEFSS foods (A: absolute intake, B: energy adjusted) pre- and post-policy by baseline levels of high-in TV advertising, excluding energy outliers.

## Data Availability

The datasets generated and/or analyzed during the current study are not publicly available, but are available under request to the corresponding author.

## Abbreviations

GOCS: Growth and Obesity Study Cohort
Kcal: Kilocalories
NFP: Nutrition Facts Panel
SSB: Sugar-sweetened beverages
TV: Television
USDA: United States Department of Agriculture
