# Supplementary material for: TV advertising and dietary intake in adolescents: a pre- and post- study of Chile’s Food Marketing Policy
Source: Int J Behav Nutr Phys Act. 2021 May 4;18:60. doi: 10.1186/s12966-021-01126-7 (PMC8097821; doi:10.1186/s12966-021-01126-7)
Supplement: Supplementary file 1 — Additional file 1: Table S1.Comparison of baseline sample (2016) with and without post-policy (2017) data. Table S2.Daily dietary intake by baseline levels of ad exposure excluding energy outliers (n=715). Table S3. Associations between policy implementation (2017 vs 2016) and consumption of overall regulated packaged foods accounting for mediation by advertising exposure excluding energy outliers (n=666; 1,332 observations). Table S4. Overall high-in consumption at baseline by levels of advertising categorized in four groups. Table S5. Overall high-in consumption at baseline by levels of advertising categorized in six groups. Table S6. Daily dietary intake by baseline levels of high-in ad exposure (in tertiles) and stratified by sex (n=721). Table S7. Associations between policy implementation (2017 vs 2016) and consumption of overall regulated packaged foods accounting for mediation by advertising exposure and stratified by sex (n=679; 1,358 observations). Table S8. Associations between policy implementation (2017 vs 2016) and consumption of overall regulated packaged foods accounting for mediation by advertising exposure and stratified by baseline levels of advertising(n=679; 1,358 observations). Figure S1. Participants (n=666; 1,332 observations) consumption of HEFSS foods (A: absolute intake, B: energy adjusted) pre- and post-policy by baseline levels of high-in TV advertising, excluding energy outliers. [file 12966_2021_1126_MOESM1_ESM.docx]

**TV Advertising and Dietary Intake in Adolescents: A Pre- and Post- study of Chile’s Food Marketing Policy**

**Online Supplemental Materials**

Melissa L. Jensen 1, 2, 3

Francesca Dillman Carpentier 4

Linda Adair 1
Camila Corvalán 5
Barry M. Popkin 1,2
Lindsey Smith Taillie 1, 2

**Author details**

1 Department of Nutrition, Gillings School of Global Public Health, University of North Carolina, Chapel Hill, NC, USA
2 Global Food Research Program, Carolina Population Center, Chapel Hill, NC, USA.
3 School of Nutrition, University of Costa Rica, San José, Costa Rica

4 Hussman School of Media and Journalism, University of North Carolina, Chapel Hill, NC, USA
5 Instituto de Nutrición y Tecnología de Alimentos, Universidad de Chile, Macul, Chile

**Correspondence to:**

Lindsey Smith Taillie
123 W Franklin St, Chapel Hill, NC, 27516

e-mail: taillie@unc.edu
Phone: (919) 962-6092
Fax: (919) 445-0740

**Table S1.** Comparison of baseline sample (2016) with and without post-policy (2017) data

| Variables | With follow-up (n=679) | | Missing follow-up (n=42) | | p-value^1^ |
| --- | --- | --- | --- | --- | --- |
|  | Mean | SD | Mean | SD |  |
| Overall high-in foods |  |  |  |  |  |
| Absolute quantity (energy) | 560.4 | 439.1 | 463.0 | 424.4 | 0.16 |
| Energy adjusted (% kcal) | 30.0 | 18.5 | 24.6 | 17.9 | 0.06 |
| Nutrients |  |  |  |  |  |
| Energy (kcal) | 1801 | 636 | 1834 | 782 | 0.74 |
| Total sugars (% energy) | 22.2 | 9.1 | 22.1 | 8.5 | 0.96 |
| Saturated fat (% energy) | 10.2 | 3.3 | 9.5 | 3.0 | 0.17 |
| Sodium (mg/1000 kcal) | 1387 | 475 | 1316 | 304 | 0.34 |
| High-in ad exposure |  |  |  |  |  |
| Any high-in product | 7.5 | 6.6 | 7.6 | 6.7 | 0.90 |
| High in calorie | 3.2 | 2.9 | 3.2 | 2.9 | 0.89 |
| High in total sugars | 4.8 | 4.3 | 4.9 | 4.3 | 0.89 |
| High in saturated fat | 1.8 | 1.7 | 1.9 | 1.8 | 0.79 |
| High in sodium | 0.7 | 0.7 | 0.7 | 0.7 | 0.93 |
| Day of week of interview (n, % week) | **558** | **82.2** | **29** | **69.0** | **0.03** |
| Child characteristics |  |  |  |  |  |
| Sex (% female) | 337 | 49.5 | 27 | 64.3 | 0.07 |
| Age (years) | 13.6 | 0.42 | 13.6 | 0.38 | 0.80 |
| Weekly screen time | 14.7 | 10.9 | 15.6 | 11.1 | 0.56 |
| Primary caregiver characteristics |  |  |  |  |  |
| Education level (n, %) |  |  |  |  |  |
| Less than high school | 201 | 29.6 | 12 | 28.6 | 0.94 |
| High school complete | 321 | 47.3 | 21 | 50.0 |  |
| More than high school | 157 | 23.1 | 9 | 21.4 |  |
| Owns home (n, % yes) | 388 | 57.1 | 22 | 52.4 | 0.55 |
| Lives with spouse or partner | 374 | 55.1 | 17 | 35.7 | 0.07 |
| ^1^ Obtained via two-sample t-test for continuous variables and X^2^ test for categorical variables. | | | | | |

**Table S2.** Daily dietary intake by baseline levels of ad exposure excluding energy outliers (n=715)

|  | Low exposure (n=241) | | Medium exposure (n=240) | | High exposure (n=240) | |
| --- | --- | --- | --- | --- | --- | --- |
|  | 0.00 min-3.1 min | | 3.1 min-9.5 min | | 9.5 min-31.8 min | |
|  | mean | SE | mean | SE | mean | SE |
| **Overall regulated packaged foods^1^** |  |  |  |  |  |  |
| Absolute energy (kcal) | 565 | 28 | 527 | 28 | 562 | 28 |
| Energy adjusted (% kcal) | 31.5 | 1.2 | 28.5 | 1.2 | 29.3 | 1.2 |
| **Nutrients** |  |  |  |  |  |  |
| Energy (kcal) | 1745 | 39 | 1803 | 38 | 1827 | 39 |
| Total sugars (grams) | 98.0 | 3.4 | 98.7 | 3.4 | 100.8 | 3.4 |
| Total sugars (% energy) | 22.5 | 0.6 | 22.0 | 0.6 | 22.1 | 0.6 |
| Saturated fat (grams) | 20.5 | 0.7 | 20.5 | 0.7 | 20.7 | 0.7 |
| Saturated fat (% energy) | 10.4 | 0.2 | 10.0 | 0.2 | 10.0 | 0.2 |
| Sodium (mg) | 2420 | 73 | 2421 | 72 | 2521 | 73 |
| Sodium (mg/1000 kcal) | 1398 | 30 | 1363 | 30 | 1388 | 30 |
| **Ready-to-eat breakfast cereals** |  |  |  |  |  |  |
| Consumers [n, (%)] | *46* | *19.2* | *30* | *12.5* | *30* | *12.8* |
| Absolute energy (kcal) | 32 | 4 | 18* | 4 | 18* | 4 |
| Energy adjusted (% kcal) | 1.8 | 0.2 | 1.2 | 0.2 | 0.9* | 0.2 |
| **Salty snacks** |  |  |  |  |  |  |
| Consumers [n, (%)] | *47* | *19.6* | *42* | *17.5* | *54* | *23.0* |
| Absolute energy (kcal) | 64 | 12 | 67 | 12 | 69 | 12 |
| Energy adjusted (% kcal) | 3.3 | 0.6 | 3.4 | 0.6 | 3.7 | 0.6 |
| **Sweets and desserts** |  |  |  |  |  |  |
| Consumers [n, (%)] | *159* | *66.3* | *159* | *66.3* | *146* | *62.1* |
| Absolute energy (kcal) | 221 | 17 | 197 | 17 | 207 | 17 |
| Energy adjusted (% kcal) | 12.2 | 0.9 | 10.7 | 0.9 | 10.9 | 0.9 |
| **Sugar-sweetened beverages** |  |  |  |  |  |  |
| Consumers [n, (%)] | *133* | *55.4* | *141* | *58.8* | *135* | *57.4* |
| Absolute energy (kcal) | 108 | 10 | 110 | 9 | 133 | 10 |
| Energy adjusted (% kcal) | 6.1 | 0.5 | 5.9 | 0.5 | 6.9 | 0.5 |
| **Milks and yogurts** |  |  |  |  |  |  |
| Consumers [n, (%)] | *49* | *20.4* | *52* | *21.7* | *38* | *16.2* |
| Absolute energy (kcal) | 30 | 5 | 35 | 5 | 26 | 5 |
| Energy adjusted (% kcal) | 1.9 | 0.3 | 2.1 | 0.3 | 1.6 | 0.3 |

^1^Model adjusted for sex, age, maternal education, home ownership, marital state, and day of week.

*P<0.05 for pairwise comparison with referent group (low exposure).

**Table S3.** Associations between policy implementation (2017 vs 2016) and consumption of overall regulated packaged foods accounting for mediation by advertising exposure excluding energy outliers (n=666; 1,332 observations)

|  | a-coefficient^2^ | | | b-coefficient | | | c'-coefficient | | | ab | | | ab/[(ab)+c’]  *100 |
| --- | --- | --- | --- | --- | --- | --- | --- | --- | --- | --- | --- | --- | --- |
|  | β | LL | UL | β | LL | UL | β | LL | UL | β | LL | UL | % |
| **Absolute quantity (kcal)^1,3^** |  |  |  |  |  |  |  |  |  |  |  |  |  |
| Any high-in ad | -4.5* | -5.6 | -3.3 | 3 | -3 | 9 | -28 | -123 | 68 | -12 | -38 | 14 | 30.3 |
| High calorie ad | -2.3* | -2.7 | -1.8 | 6 | -7 | 20 | -26 | -123 | 71 | -14 | -45 | 17 | 35.3 |
| High sugar ad | -2.9* | -3.6 | -2.2 | 3 | -6 | 13 | -30 | -125 | 66 | -10 | -37 | 17 | 24.9 |
| High fat ad | -1.3* | -1.5 | -1.0 | 13 | -11 | 37 | -23 | -120 | 74 | -16 | -47 | 15 | 41.3 |
| High sodium ad | -0.1* | -0.3 | 0.0 | 25 | -23 | 73 | -36 | -128 | 56 | -4 | -11 | 4 | 9.2 |
| **Energy adjusted (% kcal)^1,4^** |  |  |  |  |  |  |  |  |  |  |  |  |  |
| Any high-in ad | -4.5* | -5.6 | -3.3 | 0.0 | -0.2 | 0.3 | -3.7 | -7.6 | 0.2 | -0.1 | -1.1 | 1.0 | 1.8 |
| High calorie ad | -2.3* | -2.7 | -1.8 | 0.1 | -0.5 | 0.6 | -3.6 | -7.6 | 0.3 | -0.1 | -1.4 | 1.1 | 3.9 |
| High sugar ad | -2.9* | -3.6 | -2.2 | 0.0 | -0.4 | 0.4 | -3.8 | -7.7 | 0.1 | 0.0 | -1.1 | 1.1 | -0.8 |
| High Fat ad | -1.3* | -1.5 | -1.0 | 0.0 | -0.9 | 1.0 | -3.7 | -7.7 | 0.2 | -0.1 | -1.3 | 1.2 | 1.7 |
| High sodium ad | -0.1* | -0.3 | 0.0 | 0.3 | -1.6 | 2.2 | -3.7 | -7.5 | 0.0 | 0.0 | -0.3 | 0.2 | 1.1 |
| ^1^ Analyses adjusted for child's sex, age, mother’s education level, marital state, home ownership, day of recall. | | | | | | | | | | | | | |
| ^2^ Expressed in minutes of advertising per week (a-coefficient reflects policy effect on advertising) | | | | | | | | | | | | | |
| ^3^Total effect of policy implementation on absolute quantity in kcal = - 39 kcal [99CI: -38, 14 ] p=0.270 | | | | | | | | | | | | | |
| ^4^Total effect of policy implementation on energy adjusted consumption of “high in” products = -3.8 % kcal [99CI: -7.5, 0.0 ] p=0.009 | | | | | | | | | | | | | |
| * p<0.01 | | | | | | | | | | | | | |

**Table S4.** Overall high-in consumption at baseline by levels of advertising categorized in four groups

| **Weekly advertising for any high-in product** | **N** | **Crude** | | | | | | **Adjusted^1^** | | | |
| --- | --- | --- | --- | --- | --- | --- | --- | --- | --- | --- | --- |
|  |  | **Kcal** | | | **Percent kcal** | | | **Kcal** | | **Pkcal** | |
|  |  | **mean** | **SE** | **median** | **mean** | **SE** | **median** | **mean** | **SE** | **mean** | **SE** |
| No advertising | 71 | 580 | 48 | 580 | 30.3 | 1.9 | 33.4 | 572 | 52 | 30.4 | 2.2 |
| 0.1-9.99 | 419 | 535 | 20 | 443 | 29.6 | 0.9 | 26.1 | 533 | 21 | 29.6 | 0.9 |
| 10-19.99 | 192 | 572 | 35 | 453 | 29.1 | 1.3 | 28.3 | 579 | 32 | 29.3 | 1.3 |
| 20 minutes or more | 39 | 635 | 81 | 523 | 32.5 | 3.1 | 32.8 | 630 | 70 | 32.1 | 3.0 |

^1^Analyses adjusted for child's sex, age, mother’s education level, marital state, home ownership, day of recall.

**Table S5.** Overall high-in consumption at baseline by levels of advertising categorized in six groups

| **Weekly advertising for any high-in product** | **N** | **Crude** | | | | | | **Adjusted^1^** | | | |
| --- | --- | --- | --- | --- | --- | --- | --- | --- | --- | --- | --- |
|  |  | **Kcal** | | | **Percent kcal** | | | **Kcal** | | **Pkcal** | |
|  |  | **mean** | **SE** | **p50** | **mean** | **SE** | **p50** | **mean** | **SE** | **mean** | **SE** |
| No advertising | 71 | 580 | 48 | 580 | 30.3 | 1.9 | 33.4 | 572 | 52 | 30.4 | 2.2 |
| 0.1-4.99 minutes | 246 | 540 | 26 | 433 | 30.7 | 1.2 | 28.0 | 536 | 28 | 30.6 | 1.2 |
| 5-9.99 minutes | 173 | 528 | 32 | 460 | 28.2 | 1.4 | 24.0 | 530 | 33 | 28.2 | 1.4 |
| 10-14.99 minutes | 130 | 525 | 39 | 421 | 27.4 | 1.5 | 27.1 | 535 | 38 | 27.7 | 1.6 |
| 15-19.99 minutes | 62 | 669 | 69 | 598 | 32.8 | 2.7 | 29.8 | 673 | 56 | 32.6 | 2.3 |
| 20 minutes or more | 39 | 635 | 81 | 523 | 32.5 | 3.1 | 32.8 | 631 | 70 | 32.1 | 3.0 |

^1^Analyses adjusted for child's sex, age, mother’s education level, marital state, home ownership, day of recall.

**Table S6.** Daily dietary intake by baseline levels of high-in ad exposure (in tertiles) and stratified by sex (n=721)

|  | **Females (n=364)** | | | | | | **Males (n=357)** | | | | | | |
| --- | --- | --- | --- | --- | --- | --- | --- | --- | --- | --- | --- | --- | --- |
|  | Low (n=122) | | Medium (n=121) | | High (n=121) | | Low (n=119) | | Medium (n=119) | | High (n=119) | |  |
|  | 0.00-4.2 min | | 4.2-10.5 min | | 10.5-29.7 min | | 0.00-2.5 min | | 2.5-8.2 min | | 8.2-31.8 min | |  |
|  | mean | SE | mean | SE | mean | SE | mean | SE | mean | SE | mean | SE |  |
| **Overall regulated packaged foods** |  |  |  |  |  |  |  |  |  |  |  |  |  |
| Absolute energy (kcal) | 550 | 39 | 487 | 39 | 552 | 39 | 616 | 41 | 468* | 41 | 657 | 41 |  |
| Energy adjusted (% kcal) | 33.6 | 1.7 | 28.7* | 1.7 | 29.5 | 1.7 | 30.4 | 1.6 | 25.3* | 1.6 | 30.8 | 1.6 |  |
| **Nutrients** |  |  |  |  |  |  |  |  |  |  |  |  |  |
| Energy (kcal) | 1620 | 56 | 1626 | 56 | 1764 | 56 | 1927 | 59 | 1838 | 58 | 2049 | 59 |  |
| Total sugars (grams) | 93.3 | 4.5 | 91.7 | 4.5 | 98.0 | 4.5 | 108.1 | 5.5 | 96.1 | 5.5 | 114.0 | 5.5 |  |
| Total sugars (% energy) | 23.3 | 0.8 | 22.8 | 0.8 | 22.0 | 0.8 | 22.0 | 0.9 | 21.0 | 0.9 | 21.9 | 0.9 |  |
| Saturated fat (grams) | 19.2 | 1.0 | 18.7 | 1.0 | 20.3 | 1.0 | 22 | 1 | 21 | 1 | 24 | 1 |  |
| Saturated fat (% energy) | 10.6 | 0.3 | 10.0 | 0.3 | 10.1 | 0.3 | 10.2 | 0.3 | 10.0 | 0.3 | 10.1 | 0.3 |  |
| Sodium (mg) | 2285 | 95 | 2069 | 95 | 2305 | 95 | 2646 | 112 | 2623 | 111 | 133 | 133 |  |
| Sodium (mg/1000 kcal) | 1418 | 45 | 1317 | 45 | 1329 | 45 | 1390 | 40 | 1433 | 40 | 1409 | 40 |  |
| **Ready-to-eat breakfast cereals** |  |  |  |  |  |  |  |  |  |  |  |  |  |
| Consumers (n, %) | *17* | *14* | *19* | *16* | *18* | *15* | *27* | *23* | *13* | *11* | *12* | *10* |  |
| Absolute energy (kcal) | 24 | 5 | 20 | 5 | 17 | 5 | 38 | 6 | 18* | 6 | 18* | 6 |  |
| Energy adjusted (% kcal) | 1.6 | 0.3 | 1.4 | 0.3 | 1.0 | 0.3 | 1.9 | 0.4 | 1.1 | 0.4 | 0.8* | 0.4 |  |
| **Salty snacks** |  |  |  |  |  |  |  |  |  |  |  |  |  |
| Consumers (n, %) | *26* | *21* | *21* | *17* | *34* | *28* | *21* | *18* | *22* | *18* | *20* | *17* |  |
| Absolute energy (kcal) | 71 | 19 | 69 | 19 | 83 | 19 | 61 | 16 | 54 | 15 | 59 | 16 |  |
| Energy adjusted (% kcal) | 3.8 | 0.9 | 3.5 | 0.9 | 4.4 | 0.9 | 2.9 | 0.7 | 3.1 | 0.7 | 2.9 | 0.7 |  |
| **Sweets and desserts** |  |  |  |  |  |  |  |  |  |  |  |  |  |
| Consumers (n, %) | *82* | *67* | *86* | *71* | *82* | *68* | *75* | *63* | *71* | *60* | *71* | *60* |  |
| Absolute energy (kcal) | 230 | 25 | 204 | 25 | 230 | 25 | 217 | 24 | 151 | 24 | 229 | 24 |  |
| Energy adjusted (% kcal) | 14.0 | 1.3 | 11.9 | 1.3 | 12.1 | 1.3 | 10.5 | 1.1 | 7.9 | 1.1 | 11.0 | 1.1 |  |
| **Sugar-sweetened beverages** |  |  |  |  |  |  |  |  |  |  |  |  |  |
| Consumers (n, %) | *64* | *52* | *73* | *60* | *65* | *54* | *70* | *59* | *64* | *54* | *77* | *65* |  |
| Absolute energy (kcal) | 90 | 11 | 99 | 11 | 105 | 11 | 131 | 15 | 114 | 15 | 168 | 15 |  |
| Energy adjusted (% kcal) | 5.7 | 0.6 | 6.1 | 0.6 | 5.7 | 0.6 | 6.5 | 0.7 | 6.1 | 0.7 | 7.6 | 0.7 |  |
| **Milks and yogurts** |  |  |  |  |  |  |  |  |  |  |  |  |  |
| Consumers (n, %) | *27* | *22* | *25* | *21* | *20* | *17* | *23* | *19* | *23* | *19* | *23* | *19* |  |
| Absolute energy (kcal) | 34 | 6 | 29 | 6 | 24 | 6 | 30 | 7 | 34 | 7 | 34 | 7 |  |
| Energy adjusted (% kcal) | 2.3 | 0.4 | 1.8 | 0.4 | 1.6 | 0.4 | 1.6 | 0.4 | 2.0 | 0.4 | 1.7 | 0.4 |  |
| Model adjusted for age, maternal education, home ownership, marital state, and day of week. *P<0.05 for pairwise comparison with referent group (low exposure). | | | | | | | | | | | | |  |

**Table S7.** Associations between policy implementation (2017 vs 2016) and consumption of overall regulated packaged foods accounting for mediation by advertising exposure and stratified by sex (n=679; 1,358 observations)

|  | a-coefficient^2^  (policy→ads) | | b-coefficient  (ads→intake) | | c'-coefficient  (policy→intake) | | ab  (mediation effect) | | | **Total effect** | | ab/[(ab)+c’] *100 |
| --- | --- | --- | --- | --- | --- | --- | --- | --- | --- | --- | --- | --- |
|  | β± SE | p | β± SE | p | β± SE | p | | β± SE | p | **β± SE** | **p** | % |
| **Absolute quantity (kcal)** |  |  |  |  |  |  | |  |  |  |  |  |
| Girls (n=337) | -4.6±0.63 | 0.00 | 2±3 | 0.54 | -103±52 | 0.05 | | -9±14 | 0.54 | **-112±50** | **0.03** | 7.8 |
| Boys (n=384) | -4.6±0.58 | 0.00 | 5±3 | 0.16 | 22±55 | 0.69 | | -22±16 | 0.17 | 0±53 | 1.00 | NA |
| **Energy adjusted (% kcal)** |  |  |  |  |  |  | |  |  |  |  |  |
| Girls (n=337) | -4.6±0.63 | 0.00 | -0.1±0.1 | 0.54 | -5±2 | 0.01 | | 0.4±0.6 | 0.54 | -5±2 | **0.02** | NA |
| Boys (n=384) | -4.6±0.58 | 0.00 | 0.1±0.1 | 0.37 | -2±2 | 0.34 | | -1±0.6 | 0.38 | -3±2 | 0.20 | 21.4 |
| ^1^Analyses adjusted for child's age, mother’s education level, marital state, home ownership, and day of recall.  ^2^ Expressed in minutes of advertising per week (a-coefficient reflects policy effect on advertising) | | | | | | | | | | | | |

**Table S8.** Associations between policy implementation (2017 vs 2016) and consumption of overall regulated packaged foods accounting for mediation by advertising exposure and stratified by baseline levels of advertising (n=679; 1,358 observations)

|  | a-coefficient^2^  (policy→ads) | | b-coefficient  (ads→intake) | | c'-coefficient  (policy→intake) | | ab  (mediation effect) | | **Total effect** | | % mediated  ab/[(ab)+c’] *100 |
| --- | --- | --- | --- | --- | --- | --- | --- | --- | --- | --- | --- |
|  | β± SE | p | β± SE | p | β± SE | p | β± SE | p | **β±SE** | **p** |  |
| **Absolute quantity (kcal)** |  |  |  |  |  |  |  |  |  |  |  |
| Low ad exposure (n=227) | 1.0±0.3 | 0.00 | -1±9 | 0.87 | -105±61 | 0.08 | -1±9 | 0.87 | -107±60 | 0.07 | 1% |
| Moderate ad exposure (n=226) | -2.9±0.4 | 0.00 | 19±8 | 0.01 | 103±65 | 0.11 | **-56±24** | **0.02** | 46±61 | 0.45 | NA |
| High ad exposure (n=226) | -10.9±0.6 | 0.00 | 3±5 | 0.02 | -40±88 | 0.65 | -37±56 | 0.51 | -78±68 | 0.25 | 48% |
| **Energy adjusted (% kcal)** |  |  |  |  |  |  |  |  |  |  |  |
| Low ad exposure (n=227) | 1.0±0.3 | 0.00 | 0.2±0.2 | 0.62 | **-8.4±2.5** | **0.00** | 0.2±0.40 | 0.63 | **-8.2±2.5** | **0.00** | NA |
| Moderate ad exposure (n=226) | -2.9±0.4 | 0.00 | 0.7±0.3 | 0.02 | 2.6±2.5 | 0.30 | **-2.0±0.90** | **0.03** | 0.7±2.4 | 0.78 | NA |
| High ad exposure (n=226) | -10.9±0.6 | 0.00 | 0.1±0.2 | 0.54 | -1.2±3.3 | 0.71 | -1.3±2.12 | 0.54 | -2.5±2.5 | 0.32 | 51% |
| ^1^Analyses adjusted for child's sex and age, mother’s education level, marital state, home ownership, and day of recall.  ^2^ Expressed in minutes of advertising per week (a-coefficient reflects policy effect on advertising) | | | | | | | | | | | |

**Figure S1.** Participants (n=666; 1,332 observations) consumption of HEFSS foods (A: absolute intake, B: energy adjusted) pre- and post-policy by baseline levels of high-in TV advertising, excluding energy outliers


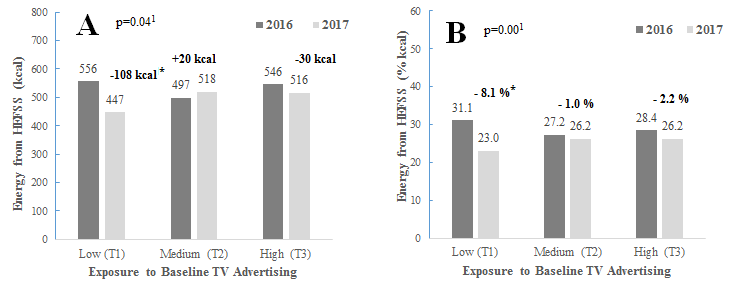


**Panel A**: absolute intake in kcal **Panel B:** energy adjusted (% kcal). Estimated using mixed model with individuals as a random effect, adjusting for study covariates. ^1^Wald test for interaction (year*baseline ad exposure).*indicates p<.05
